# Editorial Department

**Published:** 1883-07

**Authors:** 


### The Bistoury.

ELMIRA, N. Y., JULY, 1883.

The BISTOURY is published Quarterly, upon the first of April, July, October and January, at Fifty Cents a year in advance.

### Editorial Department.

VOL. XX, NO. 2 is correct. The printer had it right on the cover, wrong on the inside, last issue.

ANOTHER NEW CONTRIBUTOR has come to remain with the BISTOURY, and judging from his initial article in this number, we may safely conclude that he is likely to be entertaining and instructive. Dr. Carr's method of attacking what our homœopathic brethren call "high dilutionists," would seem convincing enough to all who have faith in the molecular theory. If the modern chemistry is correct, then, certainly cannot the Doctor's figures be disputed.

DESERVED APPOINTMENT.—We are glad to see that Dr. Lewis M. Eastman, of Baltimore, Md., has been appointed "Professor of Microscopy" in the Baltimore Medical College. No better appointment could possibly have been made. Dr. Eastman has long been recognized, by microscopists all over our land, as a very skillful manipulator of the tube, an excellent histologist and an expert in the preparation of microscopic objects. The college has done well in securing his services in lecturing upon these departments, in which he is so well versed.

THE "HEALING ART" in the good olden time, it would seem, consisted simply of the few very general and very vague operations of bleeding, sweating, purging, vomiting, salivating, stupefying, stimulating or blistering the patient. This practice evoked the scorching satire of Moliére, upon the dogmatism of the profession, in his "*Malade Imaginaire.*" His keen genius was a monument of wit and comedy of a most humiliating character to all superficial and truth-dreading followers of Æsculapius.

RAIN, RAIN!—Certainly, not within the memory of the "oldest inhabitant," have we heretofore had such a superabundance of rain as during the past month of June. While it has been excellent for the grass, according to the farmer's view of the matter, it has been very damaging to wheat and tobacco. The first week in July has been equally wet, and we hear much complaint from the Chemung County farmers thereat. "Give us dry weather for a while, or our wheat, tobacco and corn will be ruined," is their exclamation.

"DIGGERINDIAN RUGS"—Our friend C., who was informed, when purchasing a genuine, old, dirty and much worn Eastern rug, that those very qualities in the fabric made it all the more valuable, decided that he would send to the far west and secure, if possible, a pair of the dilapidated and execrably filthy blankets of the Digger Indians, christen them as above, and match them in value, ugliness and filthiness, against the more pretentious sort that come from the far East. And why not? They certainly would represent all the qualities obtained in many of the Persian rugs, and be quite as nasty.

THE ELMIRA MORNING HERALD will have made its debut by the time this number of the BISTOURY reaches our readers.

It has been said that there is no room for another morning daily in this city. So has it also been said that no more room existed here, as elsewhere, for a new doctor. But we have observed that if the doctor proved to be a capable, energetic and faithful one, he always succeeded and thrived. So it will be, we conclude, with the new morning paper. If it is so conducted as to demand our patronage—by reason of the ability with which it is edited—we will all buy it, we can not help ourselves, we must have it. Then, when it is announced that Hon. Charles G. Fairman is to conduct the editorial management, and Ausburn Towner is to become the city editor, we see at once that the *Morning Herald* will *make* room for itself. We wish the new daily success—as do we also our old stand-by, the ever-reliable *Advertiser*, whose influence for good will be by no means curtailed through the introduction of the *Herald.* May a wholesome spirit of rivalry spring up among our two morning journals as to which shall furnish the best newspaper.

OUR AMPHIUMA.—Would you believe it? But that amiable creature of which we wrote in our last number, and from which we had abstracted millions upon millions of blood-corpuscles, after patiently submitting to having his tail incised and excised upon numerous occasions, declined further to devote his caudal appendage to scientific experimentation, even if he did carry therein the largest blood-corpuscles known to man. He got weary of it, quit the tank in which he luxuriated for the past three months, and doubtless, 'ere this, is far upon his way to his native haunts in the swamps of Louisiana, where he is likely to exhibit his sore tail to his relatives and friends as a warning to keep out of sight of those men who are accredited with handling the "tube."

---

THE DUKES CASE.—We are accredited with being a law abiding people, sacrificing much to vindicate its supremacy, in giving criminals every chance that the ingenuity of counsel and the intricacies of the law offer. This is well, for popular impressions are not always correct and it is said to be better that ten guilty persons should escape than one innocent one suffer.

Occasionally, however, the jury system fails (and it is growing to be a question in this country whether jury trials are adequate to our wants) to carry out the true intent and meaning of the law, when public feeling is outraged by glaring inconsistency. It is then that in some communities Judge Lynch's method is adopted, and in others, personal chastisement and vengeance supplies the remedy.

We do not advocate either, but in a case like that of Dukes', we must close our eyes and withhold condemnation.

---

"JIM."—About six years since a boy brought us a wing-tipped Summer Duck (*Anas Sponsa*). We dressed the wound, but the wing was too much injured to be of further use. The duck soon became domesticated and was a favorite with the residents and patients at the Institute. He, too, had his favorites and welcomed them by a peculiar whistle, or by running after them and catching their dresses in his bill. "Jim," so we named him, went through, at the proper seasons, all the unpleasant features of moulting. At times he was as ugly and bedraggled as could possibly be imagined, and then he would put on his beautiful plumage and be the wonder and admiration of all. He has been immortalized on canvass and more than one of his counterfeits "in little" adorn the walls of those who seek the beautiful.

"Jim" had several mates of his own species but they did not survive captivity. Amongst the pigeons of the neighboring dove cote he would at times select a favorite, and at feeding time he would drive others away from the choice piles of feed that his associate might have a monopoly. Strange as it may seem, the pigeon would soon become attached to its friend and would leave the cote to roost in the greenhouse with "Jim."

Poor "Jim" is dead. He has been taken from us, and if there is an especial place for Good Ducks he certainly is there, but his favorite pigeon remains and mourns, wandering around thro' the day and returning at night to its lonely roost in the greenhouse, refusing to associate with other of his kind.

Poor "Jim" and poor pigeon; they are types of what may be in store for any of us. We gather around us something to love and the time comes, all too soon, when we must leave, or be left to mourn.

---

BART'S EXPERIMENT.—Bart, the janitor, was told to empty the oxygen and the hydrogen bags of their gases, the small quantity that remained in them after having burned the oxy-hydrogen light the night previously. He was directed to spread them out on the verandah floor and to carefully press out all the remaining gas with his hands, then to stop the cocks and hang them up in their accustomed places in the laboratory.

Bart is a very neat man. He likes to do every thing well and thoroughly; so, having emptied the hydrogen bag, he applied a match to the nozzle to be sure that no gas remained therein. That succeeded well enough, but when he made the same experiment with the oxygen bag, the result was quite different from what he expected.

Seated in our quiet study, we heard a terrific explosion outside—the windows rattled, house trembled and patients screamed. We were quickly on the spot to find Bart flat on his back on the lawn, with fragments of the oxygen bag about him. He had applied the match to that nozzle also with the result already detailed. Bart was pale and trembling, looking at the wreck about him in a dazed sort of way, not fully comprehending what had happened. He would like to know what the results of exploding a *full* bag of oxygen would be,* if an empty one can raise such a commotion as that! He is quite willing that other hands shall apply the match while he watches the procedure from a neighboring county by means of a telescope.

---

PRISON DISCIPLINE.—Several articles have appeared in the *Daily News*, of Newport, R. I., on this subject, and favorable comments thereon are in the *Century*, for July.

The text upon which the articles are written is comprised in one sentence of the *Century* comments, viz.: "Let criminals learn the value "of social order by giving them interests in "prison, which will demonstrate to them the "value of social order."

The plea is, that prisoners should have an opportunity of setting up business on their own account in prison, of acquiring wealth if they can earn it, supporting themselves when they can; have a good time generally in prison until their sentence shall expire and be entitled to their savings to start the world anew. It is claimed that by this plan they will learn to appreciate liberty and the value of laws for general good.

This seems to us but another link of the overstrained chain of so-called philanthropy which has become fashionable in the past twenty-five years; and under which the "Apaches" of civilization trespass with impunity on the rights and property of good citizens. Loop-holes for the scoundrel and thief to crawl through the meshes of the law are so abundant that conviction and sentence seem next to impossible, and now we find advocates for a system which will render prison life really more desirable than struggling with poverty in that seeming liberty which a poor man with a large family has outside the bars.

There are no men or women so low in life, that fail to recognize the advantages of honesty and frugality as set before them. The attempt to make prisons and penitentiaries anything more than places of detention and punishment has been met in almost every instance with signal failure. Under the philanthropic ideas and agitations of the present century crime has increased and murder and rapine stalk in our midst, oftentimes bold faced, until public opinion becomes such that the people take the law in their own hands.

Is it not about time to try the other tack? Loosely administered laws and comfortable prison quarters have been unsuccessful; perhaps the whipping post and other prison horrors may work a change.

We are inclined to think that then the punishment would enter as a factor in the contemplated crime, and instead of being incentive as a favorable change from the dens of vice outside, it would become preventive from the fear of corporal punishment and treadmill exercise, under scanty diet and uncomfortable quarters.

Of course this line of argument will be shocking to many tender-hearted good people who believe fully in human nature, but to those who realize the existence of a criminal class whose time is spent between the prison and depredations on the public, and who are callous to all teachings of good, some change seems necessary which will render the law paramount and its punishments to be feared.

Perhaps the time will come when civilization will be obliged to demand that the "Apaches" of the east shall be given up to extermination, as are their western brethren. If criminals are to be petted instead of punished, encouraged instead of crushed out, such a period is not far distant.

---

THE TRADE DOLLAR.—Here is a queer situation of affairs. Congress, in its infinite wisdom, has permitted to be coined a certain silver piece, supposed heretofore to have been money, and known to those fortunate enough to gain possession of them, as the "trade dollar." Upon the face of the coin we are certified that it contains 420 honest grains of silver, 900 fine. It bears also the great American bird, who, by some mysterious means, holds a banner aloft, bearing the patriotic motto, "*E Pluribus Unum.*" In his right, great, brawny talon he holds a bundle of arrows, as though ready to punch an eye out for any fellow that don't believe in said dollar. In the left, a bunch of hickory limbs, doubtless intended to thrash the chap courageous enough to doubt the legend below, to wit: "Trade Dollar." Then, to further deceive one into believing it to be a good and honest dollar, we have the legend over all, "United States of America." To make the proof still stronger that it is really what it pretends to be, we have on the obverse our dear old girl—the Goddess of Liberty, holding a pretty posy in her right hand, while her left grasps her dainty bustle, which is inscribed, "Liberty." She is seated on a cotton bale, with a sheaf of wheat for a back rest, and seems to be in an uncomfortable attitude, either from the cord on the bale chafing her, or from her knowledge of the fact that she is lending her presence and sanction to a swindle. She fortifies herself behind the hope "In God we Trust," thus "giving herself away"—as the boys express it—before we had any idea of her participating in such a piece of roguery.

Then, we have another dollar made of silver, without any declaration as to the number of grains of the precious metal therein contained, but which the scales declare to be just 412½. This dollar bears the face of a Dutch or French cook, which is palmed off for our beautiful goddess; a sickly and half starved crow, with wings extended in alarm over his position, takes the place of our courageous bird—the eagle. He has his bundle of arrows pointed in an opposite direction from that of our real bird, on the other coin, as though he did not want to hit anybody that did not believe his proclamation of his heralding a real silver dollar. Now, look at the injustice of the affair. The trade dollar, being a heavier one by 7½ grains, and more comely and beautiful, by a blamed sight, has been degraded by a lot of money dealers who have contrived together to boycott it out of circulation. They have fixed a price upon it, and are so obliging as to be willing to receive it at 85 cents. This loss of fifteen cents on the dollar, will be suffered by the poorer classes—those least able to bear it; so, when the bankers have purchased them all at this reduction, they can sell them to the government for their weight value as bullion and take their pay in the Bland dollar, making by the operation, 7½ grains of silver on every dollar, in addition to the fifteen cents discount at which they purchase them.

Is it not a shame that any coin bearing the imprint of the United States of America upon it, should not pass in our own country for its face value? Are there not enough patriotic Congressmen remaining to rescue our American girl and bird from the humiliating position which they occupy on the Trade Dollar? This certainly would seem to be a suitable subject upon which our statesmen might occupy their unemployed brains for a short season. It can be done. Just remove the inscription on the girl's bustle, from "Liberty," to "Legal Tender," when, presto, we have a prettier and more valuable coin in our Trade Dollar than the miserably, English designed one known as the Bland Dollar.

---

THE ADIRONDACKS.—Contrary to our usual custom—one from which we have not deviated in fifteen years—we did not go into camp at Bodines, this year, but instead thereof, made a pilgrimage to new grounds and fresher scenes.

We had heard much of a fishing resort in the Adirondacks, known as State Dam, and where the trout were said to be as plentiful as the mosquitoes and black flies. This assurance, on the part of those competent to know, decided us to visit the spot, particularly since we had inspected the swollen, inflamed and liberally speckled physiognomies of those capable and expert anglers, who had just from thence returned to convey to us this cheerful intelligence. The party consisted of five—three of them belonging to the old Bodines party—a merry, happy coterié, that met at the D. L. & W. railway station, at five o'clock one Monday morning, with excursion tickets for Malone—the home of ex-Vice-President Wheeler—located on the borders of the Adirondack regions. A detailed account of how we reached this pretty little village, after an all day ride (in which we changed cars five times, and took our meals "on the wing"), would be as vexatious to read about, as it was for us to endure. Summed up briefly, it was about as follows: Left Elmira on the Delaware, Lackawanna & Western Railroad at 5:20 A. M. Arrived in Binghamton at 7:30 where we took breakfast at the Lewis House. Took the train departing 8:30, on Rome, Watertown & Ogdensburg R. R., for Watertown; arrived there at 4:30, changing cars for DeKalb Junction, which was reached at 7, and where we had ten minutes in which to bolt a supper, telegraphed for from the last stopping place. Thence to Norwood. Left latter place at 8:25 on Ogdensburg & Lake Champlain R. R., destination, Malone. Passed through the picturesque little Pottsdam, which seemed to be struggling in an attempt to straddle two streams, one the Racquet River, filled with floating logs, saw-dust, slabs, and now and then a house or mill on stilts. What the name of the other swift and black stream is named, we did not have time to enquire. *En route* we encountered a communicative sort of chap who promptly informed us that he was the editor and proprietor of a certain weekly newspaper published at Gouverneur, a little village hard by. It would have required more than his unsupported declaration to have convinced us that he was entitled to the distinction implied to be his due by reason of his vocation, for his garb betokened the wearer to be a besotted tramp, or perhaps an impecunious Texas greaser. His verbs, adverbs and adjectives were thrown together in a reckless sort of way, having no affinity one for the other, while his sentences —usually designated, slang—were of the grotesque order. He occupied a seat immediately behind us, and was laboring to convince an acquaintance that he had better abandon the rival paper and take his "Ring-tail rorer," that gave more news to the square inch than any other paper in christendom. That his paper was conducted on a "high moral principle," was manifest when he declared that the aforesaid "Ring-tail roarer" was determined to support the candidate in the next election who exhibited the longest purse and "came down the handsomest." We parted company with him when the train stopped at Malone station, his last expressed purpose being to "strike the landlord for a night's lodging, if he'd receive pay in a column advertisement in the R. T. R."

After a refreshing sleep in comfortable apartments at the Ferguson House, in Malone, and a generous breakfast at an early hour next morning, we found a Concord coach, drawn by four horses, awaiting us at the door. This establishment had been engaged for our transportation into the wilderness, a week previously, so that no delay occurred in getting early upon the road. The distance from Malone to State Dam is sixteen miles, and over a hilly and rough country, that loses none of its attractiveness on that account. We preferred a seat on top of the vehicle, with the driver, where we enjoyed the exhilerating mountain air, the delightful views, while the interstices were admirably filled by the animated conversation of the amiable Jehu who guided the rocking, tumbling, but easy-riding conveyance over the neglected roads.

Along the entire route, on both sides of the way, on hill and in dale, were we confronted with standing hop poles, lined so precisely, and at such regular intervals, as to weary the eye with the mathematical exactness of the monstrous undertaking. Climbing the hills until the highest altitude on the journey was reached, and looking far to the rear, in the direction from which we came, we saw, seemingly along the very foot of the blue range of mountains, a bright, silvery thread that marked the pathway of the St. Lawrence River. In every direction, reaching off into vast distances, range after range of well timbered mountains were seen, varying in color from the ever changing tones of green and yellow, to the misty blue and gray of the remotest peaks. Deep down in the valley, winding about in a forest of gray hop poles, shone and sparkled the black waters of the Salmon River where it dashed against and over the huge boulders and rocks that had found a secure anchorage within its bosom. Descending suddenly from the summit of the mountain that afforded us this admirable outlook, we crossed a sandy, deserted plain, long since abandoned for purposes of agriculture, even by the hop-planters, we turned abruptly to the right, crossed the Salmon River over a rickety bridge and dove immediately into the swampy wilderness that borders the chain of lakes and streams of the Adriondack region. It was then that we realized the benefits to be derived from making such an excurson in a Concord coach.

The road was built through a tamarach swamp, and of the character known as corduroy. In spots, the saplings composing the road-bed had disappeared beneath the black mud, to find more secure soundings in the depths below. When the front wheels of the coach buried themselves to the hub in these sloughs, the inmates of the coach would all assemble, with one consent, precipitately, in a huddle, on the front seat, only to vary the posturing on the hind seat when the front and rear wheels changed position. Had the interior been lined with wood instead of soft cushions, the attempt of the passengers to make an opening through it with their heads and elbows would have been disastrous to the comeliness of the vehicle. We saw along the road side, wherever a little mound peeped above the surface of the water, several varieties of orchids in full bloom. The trilliums and columbines were also plentiful, and the dogwood and wild cherry blossoms lighted up the dark interior of the otherwise dismal swamp. The frogs and newts were piping their shrill notes by the way, while now and then the musical note of the wood-robin was heard in hidden recesses of the forest. So we plodded along—now in a chuck-hole, then rocking over the undulating surfaces of the buried saplings until interest in the scene disappeared as the backaches from the constant bracing against the rocking and tilting of the coach became more manifest. Suddenly we emerged from the wilderness into an open space, turned sharply to the left, and before we were aware of it, the coach stopped in front of a structure known as the "State Dam Hotel."

We did not dismount from our elevated seat, but sat quietly taking in our surroundings, while the Jehu released his four-in-hand from the vehicle. In front of the house was a grassy, gradually descending lawn, with here and there a ponderous boulder splitting the sod and protruding therefrom in the most inconvenient situation for such a front yard ornament. A hundred yards down the lawn, was the shore of the lake—if a mill pond full of dead hemlocks and upturned stumps could be dignified with such a name—through and into which poured the dark waters of the Salmon River. Further on, perhaps five miles away, was outlined against the sky the double humped mountain known as the "camel's back." Nearer by, and to our left, the Sugar Loaf mountain; then, nearer still, arranged about the little valley were numerous mountains of varying degree of beauty and outline.

The house itself—the only one within several miles of this spot—is neither a handsome nor a pretentious structure, but quite good enough and sufficiently well kept for the purposes intended. The doors and windows were screened with mosquito netting, upon the outside of which danced and gyrated millions of mosquitoes and black flies. The only safety from their irritating sting was to smoke yourself as does the butcher hams and sausages, in the eye-blinding "smudge." Just over a little knoll, to the right, close by the house, is the dam, a high structure built by the State, by aid of which the natives flush logs to the mills further down the stream.

At the time of our visitation, the water was very high and spread over the "meadows"—that portion of the low lands that is exhibited to view when the water follows and is confined within the channel. It was here we found the trout—out grazing, as the guide expressed it—and where we succeeded in picking up fine ones—with the fly—in sufficient quantities to make the sport lively and exhilerating. Boat fishing, or fishing from a boat, rather, has its disadvantages. You do not feel so secure in your footing as when landing a large trout from the shore. Then, you are continually raising your fish in such unexpected places and at inopportune moments. In brook fishing, a sunken log, a submerged, mossy rock, or a tuft of grass, indicates to the fisherman a suitable abiding place for a trout, so that he approaches the spot with earnest expectancy—throws his flies more deftly, trails them more carefully and is prepared to give the required jerk to fasten the fish upon his hook, upon the slightest demonstration to indicate that he is rising toward the fly. On the pond or lake, one place seems to be quite as good as another. You throw everywhere and anywhere, not knowing when to expect a response to your efforts, so that you are often surprised with a rise that might have resulted in a captured fish had your strike been made at the proper moment.

The pond and channel at State Dam are full of fish that are of fair size. So is Ragged Lake, Ragged River, Charley Lake, Indian Lake, Pompadore Lake and Salmon River, all within easy reach of the house.

For those patient anglers who are willing to fish with a worm, this territory is superb. We would recommend all such to carry bait in with them; for worms were quoted at fifty cents a quart, and exceedingly scarce and difficult to be secured even at that price.

The fly-fishermen of the party caught the fewest fish, but by far the largest ones—as they deserved to. Let us warn those adventurers who may go to this favored spot, through our recommendation of it, that there be trout and mosquitoes; trout and black flies; trout and a wearisome journey; trout and expensive fishing for them; trout and worms, $2 a gallon!

The price of board, $2 per day. Use of boat with "guide," $2.50 a day. The guide is really an unnecessary adjunct. There is no place to "guide" you. You could find your way from any pond or stream in the neighborhood with only half an eye. The charge of $2.50, for a man to row you about in a boat, is too much. It should be cheaper. We will also mildly suggest that the very obliging landlord should not permit his guests to purchase bait—such a common, ugly, wiggling bait too, as a worm—particularly when the market price is quoted as high as $2 per gallon. Just imagine the appearance of a solid gallon of angle worms poured into a heap, on the sward and charging a fisherman $2 for them!

Finally, Mr. Lowe keeps a very good house with the facilites he has at hand. Ladies *can* and *do* stop there—if they can endure "roughing it." The elevation is high, air pure, fishing abundant, ride picturesque and wearisome.

Perhaps we may go again. We will think about it.

